# SARS-CoV-2 spike protein reduces burst activities in neurons measured by micro-electrode arrays

**DOI:** 10.1097/MS9.0000000000000950

**Published:** 2023-06-10

**Authors:** Melanie Salvador, Noah Tseng, Camdon Park, Grace Williams, Arianne Vethan, Grant Thomas, John Baker, Joseph Hemry, Emma Hammond, Paige Freeburg, Guan-Wen Chou, Nick Taylor, Yi-Fan Lu

**Affiliations:** a Biology Department, Westmont College, California; b Department of Computer Science, North Carolina State University, North Carolina, USA

**Keywords:** SARS-CoV-2, spike protein, micro-electrode arrays, neuronal bursts, receptor binding domain

## Abstract

Severe acute respiratory syndrome-coronavirus-2 (SARS-CoV-2) caused a large-scale global pandemic between 2020 and 2022. Despite efforts to understand its biological and pathogenic mechanisms, the viral impact on the neurological systems remains unclear. The main goal of this study was to quantify the neurological phenotypes induced by the SARS-CoV-2 spike protein in neurons, as measured by *in-vitro* multiwell micro-electrode arrays (MEAs).

**Materials and methods:**

The authors extracted the whole-brain neurons from the newborn P1 mice and plated them on multiwell MEAs and administered purified recombinant spike proteins (both S1 and S2 subunits) from the SARS-CoV-2 virus. The signals from the MEAs were transmitted from an amplifier to a high-performance computer for recording and analysis using an in-house developed algorithm to quantify neuronal phenotypes.

**Results:**

Primary among the phenotypic features analyzed, we discovered that neuronal treatment with spike 1 protein (S1) protein from SARS-CoV-2 decreased the mean burst numbers observed on each electrode, an effect that could be rescued with an anti-S1 antibody. Conversely, this mean burst number decrease was not observed with spike 2 protein (S2) treatment. Finally, our data strongly suggest that the receptor binding domain of S1 is responsible for the reduction in neuronal burst activity.

**Conclusion:**

Overall, our results strongly indicate that spike proteins may play an important role in altering neuronal phenotypes, specifically the burst patterns, when neurons are exposed during early development.

## Introduction

Highlights

- We quantified neurological phenotypes induced by severe acute respiratory syndrome-coronavirus-2spike protein by *in-vitro* multiwell micro-electrode arrays.
- The S1 subunit of the spike protein reduced the mean number of bursts on each electrode.
- The reduction of the mean number of bursts was only observed when the neurons were treated on day 0, and mature neurons were not affected by S1.
- The reduction of the mean number of bursts was rescued by a human monoclonal anti-S1 antibody that targets the receptor binding domain.
- The receptor binding domain alone reduced the mean number of bursts in neurons.

Severe acute respiratory syndrome-coronavirus-2 (SARS-CoV-2) is the pathogenic agent responsible for the coronavirus disease 19 (COVID-19) pandemic. SARS-CoV-2 shares many common features with SARS-CoV, which also caused a brief global pandemic in 2002, leading many to believe they share a common ancestry^1^. In the more recent SARS-CoV-2 pandemic, patients hospitalized with COVID-19 often exhibited neurological symptoms including ataxia, impaired consciousness, neuralgia, and further musculoskeletal symptoms^2^. In some cases, autoantibodies against the nervous system were reported after SARS-CoV-2 infection^3,^ and may contribute to the neurological symptoms associated with COVID-19. The SARS-CoV-2 structure primarily consists of the membrane (M), envelope (E), spike (S), and nucleocapsid (N) proteins^4^. This study was primarily focused on the effects of the S protein, which assists in the attachment and entry of the virus into the host cell^5^. The S protein is comprised of two subunits (S1 and S2), each with its own function^6^. The S1 subunit is responsible for receptor binding, and the S2 subunit facilitates membrane fusion^7,8^. The protein is structured as a trimer [three receptor binding domains (RBD)], with the SARS-CoV-2 strain enabling the trimeric structure to open a pathway via pneumocytes or host cells^9^. The S proteins of SARS-CoV-2 and SARS-CoV share a sequence identity of 77%, with the predominant difference presenting in the overall 3D structure and the specific binding affinity between the SARS-CoV-2 S protein and host angiotensin-converting enzyme 2 (ACE2) receptors^2^.

The S1 subunit binds to the ACE2 receptor, which can be found on the surface of intestinal and lung cells^10^. Within the brain, ACE2 is expressed predominantly in the brainstem and other regions focused specifically on regulating cardiovascular function and blood pressure^11^. The proposed mechanism for viral attachment involves several steps that take advantage of two cleaving sites on the S protein^12^. One is located between the S1 and S2 subunit, and the other is on the S2 subunit^13^. In the first step, a proprotein convertase, predominantly furin, cleaves at the S1/S2 cleavage site^14^. It may be noted that this cleavage is specific to SARS-CoV-2 and does not occur in the original SARS virus, and likely contributes to the higher infectious properties of SARS-CoV-2 relative to the original SARS strain^5^. This cleavage destabilizes the S protein, increasing its affinity to bind to ACE2^15,16^. A D614G mutation allows rotation at that location to adopt the open conformation so that the binding site is exposed^17^. Finally, cleavage at the S2 binding site performed by transmembrane protease serine 2 results in membrane fusion^18^. Here, three previously buried hydrophobic fusion peptides in S2 become exposed and insert into the target host membrane, inducing membrane fusion^8^.

To further understand the effects of SARS-CoV-2 on the neural system, we utilized multiwell micro-electrode arrays (MEAs) to assess the neurological phenotypes *in-vitro*. MEAs contain arrays of electrodes that detect local field potentials that are generated by electrical signals generated by electrically active cell types, such as neurons or cardiomyocytes^19,20^. In this study, multiwell MEAs that contained 24 wells, each with a 4-by-4 electrode array (16 electrodes per well), were used to assess the impacts of SARS-CoV-2 spike proteins on neurons. Utilization of the MEA allows for the assessment of neuronal activity through the detection of changes in extracellular field potentials, which reflect the intrinsic action potentials of cultured neurons. The MEA possesses the ability to analyze the electric signals of a population of neurons for an extended period of time (120 000 cells for 2 weeks in the case of this experiment), exceeding the capability of a patch-clamp experiment, which only observes individual cells. The prolonged measurement over a population of neurons is an excellent means of determining the group behavior without constant perturbation, while also being analogous, although not identical, to that of the activities of the brain^21^. MEAs have been extensively used to assess the neurotoxicity of compounds and their effect on the behavior of neurons^22–24^.

In this study, we utilized MEA as an objective and non-invasive means of modeling the effects of spike proteins on neurological phenotypes under various conditions. This study may put forth a better understanding of the molecular mechanism of how the SARS-CoV-2 virion influences the neuronal system. Our study shows that the S1 protein reduces the formation of robust bursts, but only when the neurons are exposed early during the developmental stage. We further demonstrate that the RBD of S1 alone is sufficient to reduce burst activities in the neuronal population. Our study offers new insight into the biology of SARS-CoV-2 and provides an encouraging platform to assess neurological damages caused by SARS-CoV-2 proteins.

## Materials and methods

### MEA plate preparation

Sterilization of a 24-well MEA plate (AutoMate Scientific) was carried out by submerging the plate in 70% ethanol for 5 min. The plate was then air-dried for three hours or until all the remaining ethanol was completely evaporated. The coating process was performed by applying poly-D-lysine (50 μl/ml) and the plate was incubated at 37° for 1 h. The plate was then washed with sterile water and air-dried before cell plating.

### Disassociation and culturing of primary mouse cortical neurons

All animal work was ethically approved by the institution. Primary mouse cortical neurons were obtained from the newborn P1 mice. 24-well MEA plates were coated prior to the seeding of disassociated neurons. Dissection and disassociation protocols were adopted and optimized from the published work of cortical culture on MEA^25^. The cerebral cortex was removed in Hank’s Balanced Salt Solution buffer at ice–cold temperatures, and was thereafter mechanically disassociated by scissors to 1–2 mm in size. The tissue was subsequently subjected to a papain and DNase mixture for 30 min in a 37°C water bath. Cells were centrifuged at 200 RCF for 5 min and thereafter titrated by a P1000 pipette tip. 120 000 cells were seeded on top of each well covered by electrodes. Plating was done in a randomized pattern to control for confounding between spatial effects on the plates.

### Cell culture and spike protein treatment

Neurobasal–Plus media (Thermo Scientific) supplied with B-27, 10 mM HEPES, 100 U/ml penicillin streptomycin, and 2 mm glutamax was used to support the development of the neuronal culture. The media was partially changed every other day to maintain the health of the culture. Recombinant SARS-CoV-2 Spike Protein, S1, and S2 subunits were obtained from RayBiotech and stored at -80°C. On the day of neuronal cell isolation, the spike proteins were diluted to the desired concentrations and added to the culture media. To neutralize the S1 protein, 50 ng of human anti-S1 antibody (ACROBiosystems) was mixed with the S1 protein and incubated at room temperature for 3 h with gentle agitation before adding it to the culture media.

### MEA plate recording and spike quantification

Spikes were detected on a 24-well MEA plate on the MED64 Presto System (AutoMate Scientific) and the MEA Symphony Software. The threshold for calling a spike from the waveform was 500% of the SD generated from the background noise. Time stamps were recorded for the spikes that surpassed the threshold. Spikelist.cvs files with the timestamp information were exported and further analyzed in the RStudio (R-project.org). The maximum resolution of the time stamps was 0.01 ms which was able to distinguish two different spikes.

### Burst definition

Bursts were defined by an in-house developed algorithm in RStudio. The threshold to define a burst was set at contentious inter-spike intervals of less than 80 ms. Qualified bursts were recorded in a text file and exported for further analysis. After a qualifying burst was determined by the algorithm, parameters such as the timestamp, duration, spike numbers, and spike frequencies in a burst were calculated and recorded. Heatmaps of the burst data were plotted in GraphPad Prism (Insightful Science, Inc.), and the density estimation and distribution graphs were generated using the density and plot functions in RStudio (R-project.org).

### Statistical tests

The mean, standard error, and 95% CI were calculated in RStudio (RStudio.org). GraphPad Prism (Insightful Science, Inc.) was used to perform statistical tests. Unpaired two-tailed *t*-tests were conducted with a significance level set at 0.05.

## Results

### Purified S1 protein reduces burst activities in neurons

**Figure 1:**
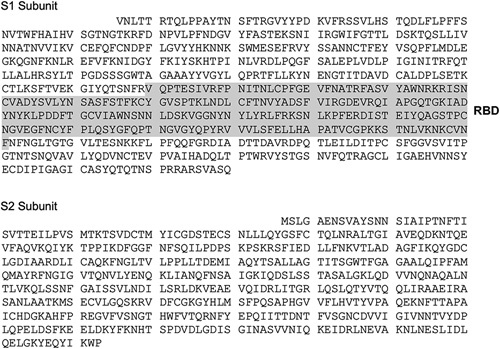
Amino acid sequences of SARS-CoV-2 Spike 1 and 2 used in this study. NCBI accession number QHD43416.

**Figure 2:**
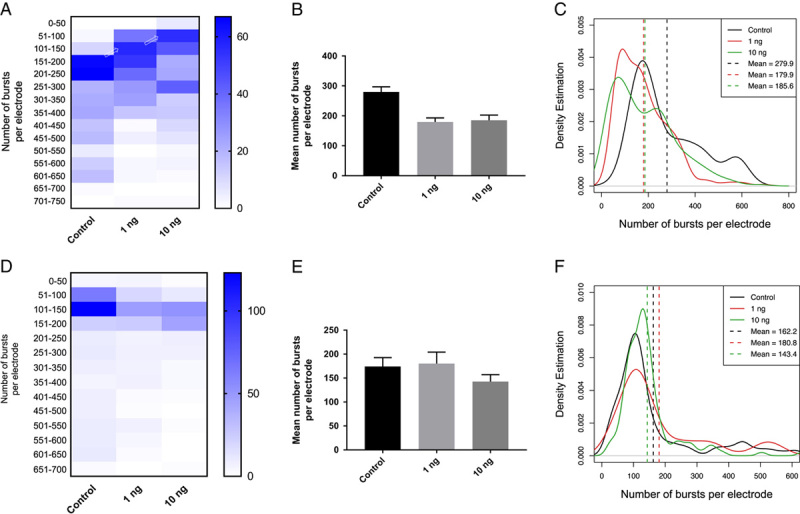
S1 reduced the number of bursts per electrode in neurons. 1 ng/ml and 10 ng/ml recombinant S1 (A–C) or S2 (D–F) were used to treat neurons on day 0. (A) The *y*-axis of the heatmap indicates the number of bursts per electrode, and the heat represents the number of electrodes (*n*). The arrows point to the shift in the number of bursts caused by 1 ng/ml or 10 ng/ml of S1. (B) The bar graph shows the mean number of bursts in each S1 treatment condition (*P*<0.0001 for both 1 ng/ml and 10 ng/ml S1 compared to the control by unpaired *t*-test) (C) The density plot shows a shift in the distribution in wells treated with S1. The dotted line indicates the mean. (D) 1 ng/ml and 10 ng/ml S2 did not reduce the number of bursts per electrode. (E) 1 ng/ml S2 did not reduce the number of bursts compared to the control (*P*=0.69), while 10 ng/ml S2 caused a borderline reduction in the number of bursts (*P*=0.03). (F) The density plot shows that both concentrations of S2 did not cause a shift in the distribution compared to the control.

Although neurological symptoms have been reported in some COVID patients, the exact mechanism of how viruses affect neuronal cells remains unclear, and thus an area of exploration. Here, we hypothesized that the spike protein of SARS-CoV-2 is responsible for the neuronal phenotypes without the need for infection by the whole infectious virion. In this study, two subunits of the spike protein (S1 and the S2), were tested separately to assess whether they induced any neurological phenotypes measured by the MEAs. We obtained recombinant SARS-CoV-2 spike protein, S1 subunit, and S2 subunits and treated neurons isolated from the P1 mice on day 0 (day 0 was defined as the day of cell preparation) (Fig. 1). Spike protein concentrations were maintained at the same level in the culture media and data from multielectrode arrays were collected over a 2-week period. We analyzed the data through an in-house developed algorithm in RStudio. Specifically, we quantified features such as the number of bursts per electrode, duration, frequency, and spike number per burst based on the treatment condition. Among all the features we investigated, we identified the number of bursts per electrode as the most prominent feature that distinguished the spike protein-treatments from the control. The S1 subunit significantly reduced the number of bursts per electrode (Table 1 and Fig. 2A–C), although this type of reduction was not as noticeable in the S2 subunit (Fig. 2D–F). Our result suggests that S1 is responsible for reducing burst activities of populations of neurons if cells are exposed early (day 0) during the developmental course.

**Table 1:** Summary statistics in this study

S1	Total number of electrodes	Mean number of bursts	Standard error	95% CI
control	281	279.9	16.7	262.5–297.3
1 ng	263	179.9	11.1	166.6–193.3
10 ng	244	185.6	11.9	168.9–202.4
S2				
control	293	162.2	10.2	138.3–186.1
1 ng	139	180.8	15.3	156.7–204.9
10 ng	133	143.4	12.4	128.8–158.0
Late treatment				
control	519	330.9	14.5	314.4–347.4
1 ng	605	419.1	17.0	403.7–434.5
10 ng	601	351.7	14.3	337.1–366.2
Antibody neutralization				
control	147	224.7	18.5	209.2–240.2
1 ng	90	179.2	18.9	165.7–192.7
1 ng + 50 ng Ab	124	238.8	21.4	216.8–260.9
RBD				
control	285	419.5	24.8	388.0–450.9
1 ng	233	283.0	18.5	257.7–308.3

### Mature neurons were not affected by S1

**Figure 3:**
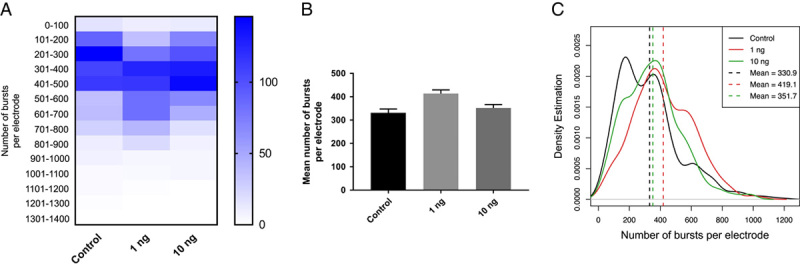
S1 did not reduce burst activities when the neurons were treated later in the developmental course. (A) Neurons were treated with 1 ng/ml S1 and 10 ng/ml, respectively, on day 12. The heatmap does not show a reduced burst number per electrode for both treatment groups compared to the control. (B) The bar graph shows that the 1 ng/ml S1 did not reduce the mean number of bursts per electrode compared to the control (*P*<0.0001 by unpaired *t*-test). 10 ng/ml S1 also did not reduce the mean number of bursts per electrode (*P*=0.07 by unpaired *t*-test). (C) The density distribution shows that both 1 ng/ml and 10 ng/ml did not cause a shift in distribution compared to the control.

Our next question was whether the S1 subunit affects mature neurons if cells are exposed to S1 later in the developmental course. The experiment was carried out with the same paradigm, except for the timeline of the S1 exposure. We treated the neurons with the same concentrations of S1 protein on day 12 and recorded the activities after the S1 exposure for 7 consecutive days. However, no noticeable reduction of burst activities was found between the S1-treated wells and the control (Fig. 3). Therefore, the data suggest that the S1 subunit only affects neurons if cells are exposed early in the developmental stage.

### Human anti-S1 antibody rescued the neuronal phenotype caused by S1

**Figure 4:**
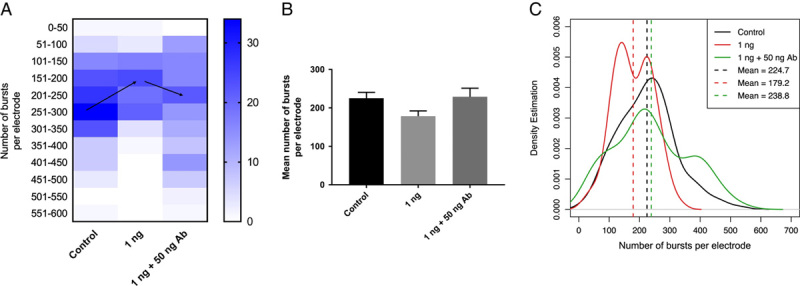
Human monoclonal anti-S1 antibody rescued the effect caused by S1. (A) 1 ng/ml S1 protein was neutralized by human anti-S1 antibody (50 ng/ml) at room temperature for 3 h before administering into the culture media at day 0. (A) The heatmap confirmed that 1 ng/ml S1 reduced the number of bursts per electrode, and the antibody treatment reverted the effect of S1 to that similar to the control (indicated by the black arrow). (B) The bar graph shows that the 1 ng/ml S1 reduced the mean number of bursts per electrode (*P*<0.0001 by unpaired *t*-test) and the neutralized S1 did not show a significant reduction (*P*=0.77 by unpaired *t*-test). (C) 1 ng/ml S1 treatment (red) caused a left skew in the distribution while the S1 + antibody treatment (green) reverted the distribution to the curve similar to the control.

To minimize the possibility that our previous observation was an artifact and the neuronal phenotype caused by S1 was in fact due to other uncharacterized factors, we conducted a rescue experiment to determine whether this neuronal phenotype can be reverted. A human monoclonal anti-S1 antibody (AS35 ACROBiosystems) was obtained and S1 was neutralized by the antibody before administering into neurons on day 0. The 1 ng/ml S1 that was neutralized by antibodies did not yield a significant reduction compared to those of the control (*P*=0.77), while the regular S1 treatment on day 0 did decrease the burst activities (Fig. 4). The data and *P* values strongly suggest that the anti-S1 antibody rescued the effect of S1 on bursting activities and the previous finding about S1’s ability to decrease neuron’s burst activities was unlikely to be an artifact. To conclude, the rescue experiment provides strong evidence that S1 was able to reduce burst activities if cells were exposed early in the developmental course.

### The RBD of S1 is responsible for reducing burst activities

**Figure 5:**
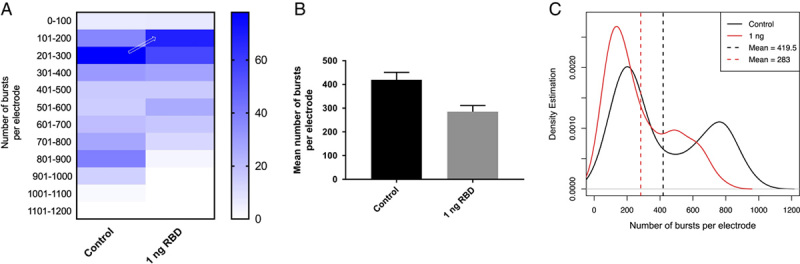
The RBD of S1 reduced the number of bursts per electrode in neurons. (A) 1 ng/ml purified RBD was used to treat neurons on day 0. The heatmap indicates a shift in the burst counts per electrode. (B) The bar graph shows a significant reduction in the mean number of bursts per electrode (*P*<0.0001 by unpaired *t*-test). (C) RBD caused a shift in distribution (the dotted line represents the mean) in the density plot.

Despite the strong evidence that S1 reduced burst count during early treatment and that the effect was reversible by the human anti-S1 antibody, it is unclear whether the full-length or a specific domain of S1 is responsible for the burst reduction. Therefore, we hypothesized that the RBD on S1 is responsible for burst reduction. We obtained purified recombinant RDB (Arg319 - Phe541) in order to test the hypothesis (Fig. 1). The experimental design followed the same experimental paradigm in which neurons were treated with 1 ng/ml RBD on day 0. RBD concentrations were then maintained at the same concentration during the whole culture period. We identified a clear reduction in burst activities that are comparable with the S1 data (Fig. 5). The result strongly suggests the fact that the RBD alone is sufficient to reduce burst activities.

## Discussion

Our study shows a clear causal relationship between the SARS-CoV-2 S1 protein and burst patterns in neuronal populations *in-vitro*, and this correlation can be rescued by antibody treatment. We further demonstrated that the RBD might be responsible for the reduction of neuronal signals. Traditionally, the understanding of the pathogenicity induced by viral diseases is thought to be due to cell damages induced by the full viral replication cycle involving attachment, entry, synthesis, and release. The full replication cycle destroys the host cells and releases more virions. In this process, the induced tissue damage may recruit immune cells and induce inflammation. Here, we show a novel aspect of SARS-CoV-2 in a multiwell MEA model: without full infection by the SARS-CoV-2 virion, S1 alone is sufficient to cause a phenotypic reduction in burst numbers. Furthermore, we show that the S1 RBD alone is sufficient to reduce burst activities, shedding new insights into the mechanism of SARS-CoV-2 that changes the cell’s phenotype beyond the traditionally understood mechanisms.

Neurons cultured in *in-vitro* MEAs are believed to be an analog to the nervous system. Although the MEAs have multiple advantages including modeling the behaviors of a population of neurons, assessing longitudinal effects of the phenotypes, and carrying out the non-invasive recordings of the electrical signals, the cells in the dish lack specialized functions that resemble areas in the brain, and thus, the cell populations should not be considered as fully functional organs or organoids. Our study aimed to capture the effect of the spike proteins in this analog system, which might be relevant to the nervous system. Whether the spike protein affects the human brain in patients requires further investigation.

The mechanism of SARS-CoV-2, specifically the role of the S protein, and its influence on the neuronal system is still unclear. Within the respiratory system, the COVID-19 infection is associated with a decreased total lung capacity, potentially resulting in overall respiratory failure and endothelial damage that disrupts pulmonary vasoregulation^22^. Connecting to brainstem affection, evidence of acute respiratory failure as a result of COVID-19 from neurotropism of the brainstem by SARS-CoV-2 also leads to pulmonary damage, with the strain invading the brain through axonal transport via the olfactory nerves^26,27^. However, there are limited findings about the neuronal impacts of SARS-CoV-2 as it travels within the lung-to-brain axis to alter neural development and respiratory function. To further understand the mechanisms and impacts of SARS-CoV-2 on the central nervous system, more research and tools are urgently required. Our study may provide the scientific community with a new instrument to study the viral impact on neurons, breaking it down to each viral protein part and assessing their influence on neurological phenotypes.

One of the more elusive aspects of COVID-19 infection is the subsequent phenomenon known as chronic post-COVID, more commonly known as long COVID^28^. Chronic post-COVID is described as prolonged symptoms after being infected by the COVID-19 virus, with symptoms lasting weeks, months, or even years after infection^29^. Chronic post-COVID is seen to be prevalent in patients who have been infected by the COVID-19 virus; patients may still develop chronic post-COVID regardless of the seriousness of the infection or the degree of treatment received^30^. There is no known mechanism for chronic post-COVID, only a theorized list of potential contributors such as post-COVID infection, organ damage, chronic inflammation, which may be caused by impaired ACE2 receptors, and traumatic effects of being in critical care^31^. Though further evaluation is needed, there seems to be an association between COVID recovery, specifically in a hospital setting, and deterioration of patient-reported quality of life. Many of the reported symptoms of chronic post-COVID are largely neurological and may require further evaluation or long-term care from medical professionals. There is no evidence that at a microbiological level, patients suffering from chronic post-COVID retain any trace of the virus within host cells. Rather, they only retain antibodies correlating to their stage of recovery^32^. Research seems to suggest that there is a correlation between the severity of the illness present and the severity of chronic post-COVID symptoms, meaning that those who were either asymptomatic or not critically infected with the virus have a better chance of not developing chronic post-COVID while those who were hospitalized are nearly twice as likely to report symptoms of chronic post-COVID^33^. Oftentimes, patients with long-term respiratory issues also complain of chronic fatigue and headaches while those with gastrointestinal issues are more likely to present elevated temperatures. Our research suggests that the spike protein of SARS-CoV-2 has the potential to induce neurological impacts that were not previously understood, which may partially explain some aspects of the chronic post-COVID symptoms associated with the central nervous system. However, further investigation is required to fully establish this direct causal relationship in patients.

The SARS-CoV-2 RBD is a common target for antibodies and vaccines^34^. SARS-CoV-2 RBD is made up of two main structures, the core and receptor binding motif (RBM). The RBM is the main fragment of the RBD that contacts the ACE2 receptor^35,36^. The RBM of the RBD contacts the underside of a small part of the ACE2 receptor to facilitate cell entry^1,37^. Here, we offered functional insight into the RBD domain of S1 and its potential importance in affecting neuronal activities. However, more investigation is required to further understand the RBD and the mechanism by which RBD alone is sufficient to affect neuronal activities.

Our study shows that by neutralizing S1, the neuronal burst activities recover to the level of the control. The human monoclonal anti-S1 antibody functions by binding to the RBD on the spike protein, preventing it from binding to ACE2^38^. Our antibody rescue experiment not only confirms the role of S1 in reducing burst activities, but also emphasizes the protective role of anti-S1 antibodies and the importance of RBD in affecting neuronal phenotypes. This finding is consistent with the clinical observations^34,39,40^. Additionally, it would be interesting to investigate whether antivirals against SARS-CoV-2 have similar protective mechanisms against S1’s impact on neurons.

## Conclusion

Overall, our study offers new insight into the understanding of the mechanism of SARS-CoV-2 spike proteins beyond their traditionally known roles of viral attachment and entry. This finding may inform important aspects about the biology of SARS-CoV-2, patient care strategies, or even future vaccine or antiviral designs.

## Ethical approval

This study does not involve patients.

## Consent

This study does not involve patients.

## Sources of funding

Westmont College Start Up Fund.

## Author contribution

Y.F.L. and J.B.: study concept or design; Y.F.L., M.S., E.H., P.F., J.B., and J.H.: data collection; Y.F.L., M.S., E.H., and P.F.: data analysis or interpretation; Y.F.L., N.T., C.P., G.W., and A.V.: writing the paper; G.T., G.W.C., and N.T.: computational framework.

## Conflicts of interest disclosure

The authors declare no conflicts of interest.

## Research registration unique identifying number (UIN)

1. Name of the registry: NA.
2. Unique Identifying number or registration ID: NA.
3. Hyperlink to your specific registration (must be publicly accessible and will be checked): NA.

## Guarantor

Yi-Fan Lu (Ph.D., Duke University).

## Provenance and peer review

Our paper was not invited.

## Data availability statement

The data is available upon reasonable request.
